# On graded classical B-2-absorbing submodules

**DOI:** 10.1016/j.heliyon.2022.e11230

**Published:** 2022-10-21

**Authors:** Khaldoun Al-Zoubi, Mohammed Ali, Mohammad Alkhatib

**Affiliations:** Department of Mathematics and Statistics, Jordan University of Science and Technology, P.O.Box 3030, Irbid 22110, Jordan

**Keywords:** Graded classical B-2-absorbing submodule, Graded classical B-prime, Graded classical 2-absorbing submodule

## Abstract

In this paper, we introduce the concept of graded classical *B*-2-absorbing submodule as a generalization of graded classical 2-absorbing submodule and we give a number of results concerning such graded modules.

## 1 Introduction

Throughout this article, we assume that ℜ is a commutative *G*-graded ring with identity and ℑ is a unitary graded ℜ-Module.

The concept of graded prime submodule was introduced by many authors, see for example [7], [13]. Several generalizations of graded prime submodules was introduced and studied, (see for example [5], [8]). In 2014, Al-Zoubi and Abu-Dawwas [1] defined a new type of graded submodules called graded 2-absorbing submodules, see also [2]. Generalizing this concept, in 2018 the notion of graded classical 2-absorbing submodules was first introduced and studied [4]. In this paper, we introduce the concept of graded classical *B*-2-absorbing submodules, and then study some important properties of this class. Here and henceforth, the notations we will use are the same ones introduced in [6]. We refer to [9], [10], [11], [12] for basic properties and more information on graded rings and graded modules.

## 2 Graded classical B-2-absorbing submodules

A nonempty subset *B* of a *G*-graded ring ℜ is said to be a multiplicatively closed subset (briefly, m.c.s) of ℜ if 0∉B, 1∈B and ab∈B for each a,b∈B. By $K\leq_{G}ℑ$, we mean that *K* is a *G*-graded submodule of ℑ.

Definition 2.1 Let B⊆h(ℜ) be a m.c.s of ℜ. A graded submodule *K* of ℑ with $(K{:}_{ℜ}ℑ)\cap B=\emptyset$ is said to be a graded classical *B*-prime (briefly, *gr*-classical *B*-prime) submodule of ℑ if there exists a fixed $s_{g}\in B$, and whenever $r_{h}t_{i}m_{j}\in K$ with $r_{h},t_{i}\in h(ℜ)$, $m_{j}\in h(ℑ)$ and h,i,j∈G, then either $s_{g}r_{h}m_{j}\in K$ or $s_{g}t_{i}m_{j}\in K$.

Definition 2.2 Let B⊆h(ℜ) be a m.c.s of ℜ. A graded submodule *K* of ℑ with $(K{:}_{ℜ}ℑ)\cap B=\emptyset$ is said to be a graded classical *B*-2-absorbing (briefly, *gr*-classical *B*-2-absorbing) submodule of ℑ if there exists a fixed $s_{g}\in B$, and whenever $r_{h}t_{i}l_{k}m_{j}\in K$ with $r_{h},t_{i},l_{k}\in h(ℜ)$, $m_{j}\in h(ℑ)$ and h,i,k,j∈G, then either $s_{g}r_{h}t_{i}m_{j}\in K$, $s_{g}r_{h}l_{k}m_{j}\in K$ or $s_{g}t_{i}l_{k}m_{j}\in K$.

Let B⊆h(ℜ) be a m.c.s of ℜ. A graded submodule *K* of ℑ with $(K{:}_{ℜ}ℑ)\cap B=\emptyset$ is said to be *a graded B*-2-absorbing (briefly, *gr*-*B*-2-absorbing) submodule of ℑ if there exists a fixed $s_{\alpha }\in B$, and whenever $r_{g}t_{h}m_{\lambda }\in K$, where $r_{g}$, $t_{h}\in h(ℜ)$ and $m_{\lambda }\in h(ℑ)$, then either $s_{\alpha }r_{g}t_{h}\in (K{:}_{ℜ}ℑ)$, $s_{\alpha }r_{g}m_{\lambda }\in K$ or $s_{\alpha }t_{h}m_{\lambda }\in K$, (see [6, Definition 2.1]).

Theorem 2.3 *Let*B⊆h(ℜ)*be a*m.c.s*of* ℜ*. If K is a gr-B-*2*-absorbing submodule of* ℑ*, then K is gr-classical B-*2*-absorbing submodule of* ℑ*.*

Proof Let *K* be a *gr*-*B*-2-absorbing submodule of ℑ. Then $(K{:}_{ℜ}ℑ)\cap B=\emptyset$. Now, let $r_{h},t_{i},l_{k}\in h(ℜ)$ and $m_{j}\in h(ℑ)$ with $r_{h}t_{i}l_{k}m_{j}\in K$ and h,i,k,j∈G. Since $l_{k}\in h(ℜ)$ and $m_{j}\in h(ℑ)$, then $l_{k}m_{j}\in h(ℑ)$. So, there is a fixed $s_{g}\in B$ such that either $s_{g}r_{h}t_{i}\in (K{:}_{ℜ}ℑ)$, $s_{g}r_{h}(l_{k}m_{j})\in K$ or $s_{g}t_{i}(l_{k}m_{j})\in K$. Thus either $s_{g}r_{h}t_{i}m_{j}\in K$, $s_{g}r_{h}l_{k}m_{j}\in K$ or $s_{g}t_{i}l_{k}m_{j}\in K$. Therefore, *K* is a *gr*-classical *B*-2-absorbing submodule of ℑ. □

A proper graded submodule *K* of ℑ is said to be a graded classical 2-absorbing (briefly, *gr*-classical 2-absorbing) submodule of ℑ if whenever r,s,t∈h(ℜ) and m∈h(ℑ) with rstm∈K, then either rsm∈K, rtm∈K or stm∈K, (see [4]).

The following result can be easily obtained. (We omit the proof.)

Theorem 2.4 *Let*$B_{1}\subseteq B_{2}\subseteq h(ℜ)$*be two*m.c.s*of* ℜ*. If K is a gr-classical* $B_{1}$*-*2*-absorbing submodule of* ℑ *such that* $(K{:}_{ℜ}ℑ)\cap B_{2}=\emptyset$*, then K is also a gr-classical* $B_{2}$*-*2*-absorbing submodule of* ℑ*. In particular, every gr-classical 2-absorbing submodule K with* $(K{:}_{ℜ}ℑ)\cap B=\phi$ *is a gr-classical B-2-absorbing.*

The converse of Theorem 2.4 is not true in general, the following example illustrates that.

Example 2.5 Let G=Z. Let ℜ=Z be a *G*-graded ring with ${ℜ}_{h}=\left\{\begin{array}{cc} Z\; & \text{if }h=0\\ 0\; & \text{otherwise} \end{array}\right\}$ and ℑ=Z×Z be a graded ℜ-module with ${ℑ}_{h}=\left\{\begin{array}{cc} Z\times \{0\}\; & \text{if }h=0\\ \{0\}\times Z\; & \text{if }h=1\\ \{0\}\times \{0\}\; & \text{otherwise} \end{array}\right\}$. Consider the graded submodule K={0}×16Z. Since $2^{3}\cdot (0,2)\in K$ and $2^{2}\cdot (0,2)\notin K$, then *K* is not *gr*-classical 2-absorbing submodule of ℑ. Now take the m.c.s. $B=\{2^{n}:n\in N\cup \{0\}\}$ and put s=2. It is easy to see that *K* is a *gr*-classical *B*-2-absorbing submodule of ℑ.

Theorem 2.6 *Let*B⊆h(ℜ)*be a*m.c.s*of* ℜ *and* $K\leq_{G}ℑ$ *such that* $(K{:}_{ℜ}ℑ)\cap B=\emptyset$*. Assume that* B⊆u(ℜ)∩h(ℜ)*, where* u(ℜ) *is the set of all units in* ℜ*. Then the following are equivalent:*

(i) *K is gr-classical B-*2*-absorbing.*
(ii) *K is gr-classical* 2*-absorbing submodule of* ℑ*.*

Proof (i)⇒(ii) Let *K* be a *gr*-classical *B*-2-absorbing submodule of ℑ and $s_{g}\in B$ be the element which satisfies the *gr*-classical *B*-2-absorbing submodule condition. Let $r_{h}t_{i}l_{k}m_{j}\in K$, where $r_{h},t_{i},l_{k}\in h(ℜ)$, $m_{j}\in h(ℑ)$ and g,h,i,k,j∈G. So we have either $s_{g}r_{h}t_{i}m_{j}\in K$, $s_{g}r_{h}l_{k}m_{j}\in K$ or $s_{g}t_{i}l_{k}m_{j}\in K$. Since *K* is graded submodule of ℑ, we get $r_{h}t_{i}m_{j}\in K$, $r_{h}l_{k}m_{j}\in K$ or $t_{i}l_{k}m_{j}\in K$. Therefore, *K* is *gr*-classical 2-absorbing submodule of ℑ.

(ii)⇒(i) See Theorem 2.4. □

A graded classical 2-absorbing submodule does not imply graded classical prime, (see [4, Example 2.4]), so Theorem 2.6 shows that graded classical *B*-2-absorbing submodule need not to be graded classical *B*-prime by taking B=u(ℜ)∩h(ℜ). A graded classical 2-absorbing submodule does not imply graded 2-absorbing, (see [4, Example 2.4]) so Theorem 2.6 shows that a graded classical *B*-2-absorbing submodule need not be graded *B*-2-absorbing submodule by taking B=u(ℜ)∩h(ℜ).

Theorem 2.7 *Let*$B_{1}\subseteq B_{2}\subseteq h(ℜ)$*be two*m.c.s*of* ℜ *and for every* $s_{g}\in B_{2}$*, there exists* $v_{l}\in h(ℜ)$ *such that* $s_{g}v_{l}\in B_{1}$*, where* g,l∈G*. If K is a gr-classical* $B_{2}$*-*2*-absorbing submodule of* ℑ*, then K is gr-classical* $B_{1}$*-*2*-absorbing submodule of* ℑ*.*

Proof Let *K* be a *gr*-classical $B_{2}$-2-absorbing submodule of ℑ. Then $(K{:}_{ℜ}ℑ)\cap B_{2}=\emptyset$, it follows that $(K{:}_{ℜ}ℑ)\cap B_{1}=\emptyset$. Now, let $r_{h}t_{i}l_{k}m_{j}\in K$ with $r_{h},t_{i},l_{k}\in h(ℜ)$, $m_{j}\in h(ℑ)$ and h,i,k,j∈G. Then there exists $s_{g}\in B_{2}$ such that either $s_{g}r_{h}t_{i}m_{j}\in K$, $s_{g}r_{h}l_{k}m_{j}\in K$ or $s_{g}t_{i}l_{k}m_{j}\in K$. Since $s_{g}\in B_{2}$, there exists $v_{m}\in h(ℜ)$ with $s_{g}v_{m}\in B_{1}$, where m∈G. Take $s^{\prime }=s_{g}v_{m}\in B_{1}$, then either $s^{\prime }r_{h}t_{i}m_{j}=(s_{g}v_{m})r_{h}t_{i}m_{j}\in K$, $s^{\prime }r_{h}l_{k}m_{j}=(s_{g}v_{m})r_{h}l_{k}m_{j}\in K$ or $s^{\prime }t_{i}l_{k}m_{j}=(s_{g}v_{m})t_{i}l_{k}m_{j}\in K$. Thus, *K* is *gr*-classical $B_{1}$-2-absorbing submodule of ℑ. □

Let B⊆h(ℜ) be a m.c.s of ℜ. The *gr*-saturation of *B* is defined by $B^{⁎}=\{s_{g}\in h(ℜ):\frac{s_{g}}{1}$ is a unit in $B^{-1}ℜ\}$. It is easy to show that $B^{⁎}$ is m.c.s of ℜ containing *B*. Also, a m.c.s B⊆h(ℜ) is *gr*-saturated set if $B=B^{⁎}$.

Theorem 2.8 *Let*B⊆h(ℜ)*be a*m.c.s*of* ℜ *and* $K\leq_{G}ℑ$*. Assume that* $B^{⁎}$ *is the gr-saturation of B. Then the following are equivalent:*

(i) *K is gr-classical B-*2*-absorbing submodule of* ℑ*.*
(ii) *K is gr-classical*$B^{⁎}$*-*2*-absorbing submodule of* ℑ*.*

Proof (i)⇒(ii) Let *K* be a *gr*-classical *B*-2-absorbing submodule of ℑ. So $(K{:}_{ℜ}ℑ)\cap B=\emptyset$. If $(K{:}_{ℜ}ℑ)\cap B^{⁎}\neq \emptyset$, then we have $r_{g}\in (K{:}_{ℜ}ℑ)\cap B^{⁎}$. Hence, $\frac{r_{g}}{1}$ is a unit homogeneous element of $B^{-1}ℜ$. Thus, there exist $t_{h}\in h(ℜ)$ and $s_{i}\in B$ such that $\frac{r_{g}}{1}\frac{t_{h}}{s_{i}}=\frac{r_{g}t_{h}}{s_{i}}=1$. So, $u_{j}s_{i}=u_{j}r_{g}t_{h}$ for some $u_{j}\in B$. Since $r_{g}\in (K{:}_{ℜ}ℑ)$, $u_{j}r_{g}t_{h}\in (K{:}_{ℜ}ℑ)$. This gives that $u_{j}r_{g}t_{h}\in (K{:}_{ℜ}ℑ)\cap B$, which is a contradiction. Since $B\subseteq B^{⁎}$, then by Theorem 2.4, *K* is *gr*-classical $B^{⁎}$-2-absorbing submodule of ℑ.

(ii)⇒(i) Suppose that *K* is a *gr*-classical $B^{⁎}$-2-absorbing submodule of ℑ, so $(K{:}_{ℜ}ℑ)\cap B^{⁎}=\emptyset$. Since $B\subseteq B^{⁎}$, then $(K{:}_{ℜ}ℑ)\cap B=\emptyset$. Now, let $a_{h}b_{i}c_{k}m_{j}\in K$, where $a_{h},b_{i},c_{k}\in h(ℜ)$, $m_{j}\in h(ℑ)$ and h,i,j,k∈G. So, there exists $s_{g}\in B^{⁎}$ with g∈G such that either $s_{g}a_{h}b_{i}m_{j}\in K$, $s_{g}a_{h}c_{k}m_{j}\in K$ or $s_{g}b_{i}c_{k}m_{j}\in K$, since *K* is *gr*-classical $B^{⁎}$-2-absorbing submodule of ℑ. As $s_{g}\in B^{⁎}$, then $\frac{s_{g}}{1}$ is a unit homogeneous element of $B^{-1}ℜ$. This leads to there exists $r_{y}\in h(ℜ)$ and $t_{z}\in B$ with $\frac{s_{g}}{1}\frac{r_{y}}{t_{z}}=1$, where y,z∈G. Hence, $v_{w}s_{g}r_{y}=v_{w}t_{z}$, for some $v_{w}\in B$ and w∈G. Now take $s^{\prime }=v_{w}t_{z}=v_{w}r_{y}s_{g}\in B$. Then either $s^{\prime }a_{h}b_{i}m_{j}=v_{w}r_{y}(s_{g}a_{h}b_{i}m_{j})\in K$, $s^{\prime }a_{h}c_{k}m_{j}=v_{w}r_{y}(s_{g}a_{h}c_{k}m_{j})\in K$ or $s^{\prime }b_{i}c_{k}m_{j}=v_{w}r_{y}(s_{g}b_{i}c_{k}m_{j})\in K$. Therefore, *K* is *gr*-classical *B*-2-absorbing submodule of ℑ. □

Theorem 2.9 *Let*B⊆h(ℜ)*be a*m.c.s*of* ℜ*. If K is a gr-classical B-*2*-absorbing submodule of* ℑ*, then* $B^{-1}K$ *is a gr-classical 2-absorbing submodule of* $B^{-1}ℑ$*.*

Proof Let $\frac{a}{s}\frac{b}{t}\frac{c}{l}\frac{m}{w}\in B^{-1}K$ with $\frac{a}{s},\frac{b}{t},\frac{c}{l}\in h(B^{-1}ℜ)$ and $\frac{m}{w}\in h(B^{-1}ℑ)$. Then (ua)bcm∈K for some u∈B. Thus there exists $s^{\prime }\in B$ with $s^{\prime }(ua)bm\in K$, $s^{\prime }(ua)cm\in K$ or $s^{\prime }bcm\in K$. This implies that $\frac{a}{s}\frac{b}{t}\frac{m}{w}=\frac{s^{\prime }uabm}{s^{\prime }ustw}\in B^{-1}K$ or $\frac{a}{s}\frac{c}{l}\frac{m}{w}=\frac{s^{\prime }uacm}{s^{\prime }uslw}\in B^{-1}K$ or $\frac{c}{l}\frac{b}{t}\frac{m}{w}=\frac{s^{\prime }ucbm}{s^{\prime }ultw}\in B^{-1}K$. Therefore, $B^{-1}K$ is *gr*-classical *B*-2-absorbing submodule of $B^{-1}ℑ$. □

Theorem 2.10 *Let*B⊆h(ℜ)*be a finite*m.c.s*of* ℜ*. If* $B^{-1}K$ *is a gr-classical* 2*-absorbing submodule of* $B^{-1}ℑ$*, then K is gr-classical B-*2*-absorbing submodule of* ℑ*.*

Proof Suppose that $B^{-1}K$ is a *gr*-classical 2-absorbing submodule of $B^{-1}ℑ$, $B=\{s_{1},s_{2},....,s_{k}\}$ and $s=s_{1}s_{2}.....s_{k}$. Let $a_{h}b_{i}c_{k}m_{j}\in K$, where $a_{h},b_{i},c_{k}\in h(ℜ)$, $m_{j}\in h(ℑ)$ and h,i,k,j∈G. Hence, $\frac{a_{h}}{1}\frac{b_{i}}{1}\frac{c_{k}}{1}\frac{m_{j}}{1}\in B^{-1}K$. Since $\frac{a_{h}}{1},\frac{b_{i}}{1},\frac{c_{k}}{1}\in h(B^{-1}ℜ),\frac{m_{j}}{1}\in h(B^{-1}ℑ)$ and $B^{-1}K$ is *gr*-classical 2-absorbing submodule of $B^{-1}ℑ$, we get either $\frac{a_{h}}{1}\frac{b_{i}}{1}\frac{m_{j}}{1}\in B^{-1}K$, $\frac{a_{h}}{1}\frac{c_{k}}{1}\frac{m_{j}}{1}\in B^{-1}K$ or $\frac{b_{i}}{1}\frac{c_{k}}{1}\frac{m_{j}}{1}\in B^{-1}K$. So, we conclude that either $sa_{h}b_{i}m_{j}\in K$, $sa_{h}c_{k}m_{j}\in K$ or $sb_{i}c_{k}m_{j}\in K$. Therefore, *K* is *gr*-classical *B*-2-absorbing submodule of ℑ. □

Theorem 2.11 *Let*B⊆h(ℜ)*be a*m.c.s*of* ℜ*,* $K\leq_{G}ℑ$ *and* $x_{m}\in h(ℑ)-K$ *with* $(K{:}_{ℜ}x_{m})\cap B=\emptyset$ *and* m∈G*. If K is gr-classical B-*2*-absorbing submodule of* ℑ*, then* $I=(K{:}_{ℜ}x_{m})$ *is graded B-*2*-absorbing ideal of* ℜ*.*

Proof Let $r_{h}t_{i}l_{k}\in (K{:}_{ℜ}x_{m})$ such that $r_{h},t_{i},l_{k}\in h(ℜ)$ and h,i,k∈G. So, $r_{h}t_{i}l_{k}x_{m}\in K$. Then there exists $s_{g}\in B$ such that $s_{g}r_{h}t_{i}x_{m}\in K$, $s_{g}r_{h}l_{k}x_{m}\in K$ or $s_{g}t_{i}l_{k}x_{m}\in K$, where g∈G. So, $s_{g}r_{h}t_{i}\in (K{:}_{ℜ}x_{m})$, $s_{g}r_{h}l_{k}\in (K{:}_{ℜ}x_{m})$ or $s_{g}t_{i}l_{k}\in (K{:}_{ℜ}x_{m})$. Hence, *I* is graded *B*-2-absorbing ideal of ℜ. □

Theorem 2.12 *Let*$ℑ=ℜx_{g}$*(for some*$x_{g}\in h(ℑ)$*) be a graded cyclic* ℜ*-module and* B⊆h(ℜ) *be a* m.c.s *of* ℜ*. Then every gr-classical B-2-absorbing submodule of* ℑ *is gr-B-*2*-absorbing submodule of* ℑ*.*

Proof Let *K* be *gr*-classical *B*-2-absorbing submodule of ℑ. Let $a_{h}b_{i}m_{j}\in K$, where $a_{h},b_{i}\in h(ℜ)$, $m_{j}\in h(ℑ)$ and h,i,j∈G. As $m_{j}\in h(ℑ)$, there exists $r_{t}\in h(ℜ)$ such that $m_{j}=r_{t}x_{g}$, where t∈G. Hence $a_{h}b_{i}r_{t}x_{g}\in K$. So there is $s_{k}\in B$ with k∈G such that either $s_{k}a_{h}b_{i}x_{g}\in K$, $s_{k}a_{h}r_{t}x_{g}\in K$ or $s_{k}b_{i}r_{t}x_{g}\in K$. Hence $s_{k}a_{h}b_{i}\in (K{:}_{ℜ}x_{g})=(K{:}_{ℜ}ℑ)$, $s_{k}a_{h}m_{j}\in K$ or $s_{k}b_{i}m_{j}\in K$. Thus, *K* is a *gr*-*B*-2-absorbing submodule of ℑ. □

Let B⊆h(ℜ) be a m.c.s of ℜ and *I* be a graded ideal of ℜ with I∩B=∅. We say that *I* is *a graded B-Prime ideal* if there exists $s_{i}\in B$ and whenever $r_{g}d_{h}\in I$ with $r_{g},d_{h}\in h(ℜ)$ and g,h∈G, then either $s_{i}r_{g}\in I$ or $s_{i}d_{h}\in I$, (see [3]).

Theorem 2.13 *Let*B⊆h(ℜ)*be a*m.c.s*of* ℜ*. If K is a gr-B-*2*-absorbing submodule of* ℑ *and* $\left(K{:}_{ℜ}ℑ\right)$ *is a graded B-prime ideal of* ℜ*, then K is a gr-classical B-prime submodule of* ℑ*.*

Proof Let *K* be a *gr*-*B*-2-absorbing submodule of ℑ and $\left(K{:}_{ℜ}ℑ\right)$ be a graded *B*-prime ideal of ℜ. Suppose that $s_{g}^{\prime }\in B$ (g∈G) is the element that makes *K* satisfies the graded *B*-2-absorbing submodule condition, and suppose that $s_{k}^{⁎}\in B$ (k∈G) makes $\left(K{:}_{ℜ}ℑ\right)$ satisfies the graded classical *B*-prime ideal condition. Fix $s_{kg}=s_{k}^{⁎}s_{g}^{\prime }\in B$, and let $r_{h}t_{i}m_{j}\in K$ with $r_{h},t_{i}\in h(ℜ)$, $m_{j}\in h(ℑ)$ and h,i,j∈G. Then either $s_{g}^{\prime }r_{h}t_{i}\in (K{:}_{ℜ}ℑ)$, $s_{g}^{\prime }r_{h}m_{j}\in K$ or $s_{g}^{\prime }t_{i}m_{j}\in K$. If one of the last two cases holds, then we are done. Assume that $s_{g}^{\prime }r_{h}t_{i}\in (K{:}_{ℜ}ℑ)$. Since $\left(K{:}_{ℜ}ℑ\right)$ is a graded *B*-prime ideal of ℜ, we get either $s_{k}^{⁎}s_{g}^{\prime }r_{h}=s_{kg}r_{h}\in (K{:}_{ℜ}ℑ)$ or $s_{k}^{⁎}s_{g}^{\prime }t_{i}=s_{kg}t_{i}\in (K{:}_{ℜ}ℑ)$. Thus $s_{kg}r_{h}m_{j}\in K$ or $s_{kg}t_{i}m_{j}\in K$. Thus, *K* is *gr*-classical *B*-prime submodule of ℑ. □

Theorem 2.14 *Let*B⊆h(ℜ)*be a*m.c.s*of* ℜ*. Then intersection of two gr-classical B-prime submodules of* ℑ *is a gr-classical B-*2*-absorbing submodule of* ℑ*.*

Proof Suppose that $N_{1},N_{2}$ are two *gr*-classical *B*-prime submodules of ℑ and $s_{g}$, $s_{h}\in B$ make $N_{1},N_{2}$ satisfy the *gr*-classical *B*-prime condition, respectively. Fix $s_{gh}=s_{g}s_{h}\in {ℜ}_{gh}$, and let $a_{t}b_{i}c_{k}m_{j}\in N_{1}\cap N_{2}$ where $a_{t},b_{i},c_{k}\in h(ℜ)$, $m_{j}\in h(ℑ)$ and t,i,j,k∈G. Then $a_{t}b_{i}c_{k}m_{j}\in N_{1}$. It follows that $s_{g}a_{t}m_{j}\in N_{1}$, $s_{g}b_{i}m_{j}\in N_{1}$ or $s_{g}c_{k}m_{j}\in N_{1}$. Similarly, we have either $s_{h}a_{t}m_{j}\in N_{2}$, $s_{h}b_{i}m_{j}\in N_{2}$ or $s_{h}c_{k}m_{j}\in N_{2}$. This implies that $s_{gh}a_{t}b_{i}m_{j}\in N_{1}\cap N_{2}$, $s_{gh}a_{t}c_{k}m_{j}\in N_{1}\cap N_{2}$ or $s_{gh}b_{i}c_{k}m_{j}\in N_{1}\cap N_{2}$. Hence, $N_{1}\cap N_{2}$ is *gr*-classical *B*-2-absorbing submodule of ℑ. □

Lemma 2.15 *Let*B⊆h(ℜ)*be a*m.c.s*of* ℜ *and N be a gr-classical B-*2*-absorbing submodule of* ℑ*. Let* $I={⨁}_{\alpha \in G}I_{\alpha }$ *be a graded ideal of* ℜ*, where* $I_{\alpha }=I\cap {ℜ}_{\alpha }$*. Then there exists* $s_{g}\in B$*, where* g∈G *such that for every* α∈G*, if* $I_{\alpha }a_{h}b_{i}m_{j}\subseteq N$ *for some* $a_{h},b_{i}\in h(ℜ)$*,* $m_{j}\in h(ℑ)$ *and* h,j,j∈G*, then either* $s_{g}I_{\alpha }a_{h}m_{j}\subseteq N$ *or* $s_{g}I_{\alpha }b_{i}m_{j}\subseteq N$ *or* $s_{g}a_{h}b_{i}m_{j}\in N$*.*

Proof Since *N* is a *gr*-classical *B*-2-absorbing submodule of ℑ, then there exists $s_{g}\in B$ such that whenever $r_{h}t_{i}l_{k}m_{j}\in N$, where $r_{h},t_{i},l_{k}\in h(ℜ)$, $m_{j}\in h(ℑ)$ and h,i,j,k∈G, we have either $s_{g}r_{h}t_{i}m_{j}\in N$, $s_{g}t_{i}l_{k}m_{j}\in N$ or $s_{g}r_{h}l_{k}m_{j}\in N$. Assume that $I_{\alpha }r_{h}t_{i}m_{j}\subseteq N$, where $I_{\alpha }=I\cap {ℜ}_{\alpha }$, $r_{h},t_{i}\in h(ℜ)$, $m_{j}\in h(ℑ)$ and h,i,j,α∈G. Suppose that $s_{g}I_{\alpha }t_{i}m_{j}⊈N$ and $s_{g}r_{h}t_{i}m_{j}\notin N$. We need to show that $s_{g}I_{\alpha }r_{h}m_{j}\subseteq N$. Let $a_{\alpha }\in I_{\alpha }$. Since $s_{g}I_{\alpha }t_{i}m_{j}⊈N$, then there exists $b_{\alpha }\in I_{\alpha }\subseteq h(ℜ)$ such that $s_{g}b_{\alpha }t_{i}m_{j}\notin N$. Since $I_{\alpha }r_{h}t_{i}m_{j}\subseteq N$, we have $b_{\alpha }r_{h}t_{i}m_{j}\in N$. Then $s_{g}r_{h}b_{\alpha }m_{j}\in N$. Since $a_{\alpha },b_{\alpha }\in I_{\alpha }$, we get $a_{\alpha }+b_{\alpha }\in I_{\alpha }$. Because $I_{\alpha }r_{h}t_{i}m_{j}\subseteq N$, $(a_{\alpha }+b_{\alpha })r_{h}t_{i}m_{j}\in N$. Then either $s_{g}(a_{\alpha }+b_{\alpha })r_{h}m_{j}\in N$ or $s_{g}(a_{\alpha }+b_{\alpha })t_{i}m_{j}\in N$. If $s_{g}(a_{\alpha }+b_{\alpha })r_{h}m_{j}=s_{g}a_{\alpha }r_{h}m_{j}+s_{g}b_{\alpha }r_{h}m_{j}\in N$, then as $s_{g}b_{\alpha }r_{h}m_{j}\in N$, it follows that $s_{g}a_{\alpha }r_{h}m_{j}\in N$. However, if $s_{g}(a_{\alpha }+b_{\alpha })t_{i}m_{j}\in N$, then by $s_{g}b_{\alpha }t_{i}m_{j}\notin N$ it follows that $s_{g}a_{\alpha }t_{i}m_{j}\notin N$. But we have $a_{\alpha }t_{i}r_{h}m_{j}\in N$, which gives $s_{g}a_{\alpha }r_{h}m_{j}\in N$. Since *N* is a *gr*-classical *B*-2-absorbing submodule of ℑ, $s_{g}a_{\alpha }t_{i}m_{j}\notin N$ and $s_{g}r_{h}t_{i}m_{j}\notin N$, then we get that $s_{g}I_{\alpha }r_{h}m_{j}\subseteq N$. □

Theorem 2.16 *Let*B⊆h(ℜ)*be a*m.c.s*of* ℜ *and N be a gr-classical B-*2*-absorbing submodule of* ℑ*. Let* $I={⨁}_{\alpha \in G}I_{\alpha }$ *and* $J={⨁}_{\beta \in G}J_{\beta }$ *be graded ideals of* ℜ *where* $I_{\alpha }=I\cap {ℜ}_{\alpha }$ *and* $J_{\beta }=J\cap {ℜ}_{\beta }$*. Then there exists* $s_{t}\in B$*, where* t∈G *such that for every* α,β∈G*, if* $I_{\alpha }J_{\beta }a_{h}m_{j}\subseteq N$ *for some* $a_{h}\in h(ℜ)$*,* $m_{j}\in h(ℑ)$ *and* h,j∈G*, then either* $s_{t}I_{\alpha }a_{h}m_{j}\subseteq N$ *or* $s_{t}I_{\alpha }J_{\beta }m_{j}\subseteq N$ *or* $s_{t}J_{\beta }a_{h}m_{j}\subseteq N$*.*

Proof Since *N* is a *gr*-classical *B*-2-absorbing submodule of ℑ, then by Lemma 2.15, for every α∈G there exists $s_{t}\in B$, where t∈G such that whenever $I_{\alpha }a_{h}b_{i}m_{j}\subseteq N$ for some $a_{h},b_{i}\in h(ℜ)$, $m_{j}\in h(ℑ)$ and h,j,j∈G, we have either $s_{t}I_{\alpha }a_{h}m_{j}\subseteq N$, $s_{t}I_{\alpha }b_{i}m_{j}\subseteq N$ or $s_{t}a_{h}b_{i}m_{j}\in N$. Let $I_{\alpha }J_{\beta }a_{h}m_{j}\subseteq N$ where $a_{h}\in h(ℜ)$, $m_{j}\in h(ℑ)$ and h,j∈G. Suppose that $s_{t}I_{\alpha }a_{h}m_{j}⊈N$ and $s_{t}J_{\beta }a_{h}m_{j}⊈N$. We are going to show that $s_{t}I_{\alpha }J_{\beta }m_{j}\subseteq N$. Let $c_{\alpha }\in I_{\alpha }$ and $d_{\beta }\in J_{\beta }$. Since $s_{t}I_{\alpha }a_{h}m_{j}⊈N$, then there exists $r_{\alpha }\in I_{\alpha }$ such that $s_{t}r_{\alpha }a_{h}m_{j}\notin N$, so $r_{\alpha }\notin (N{:}_{ℜ}s_{t}a_{h}m_{j})$ which means that $r_{\alpha }\in I_{\alpha }-(N{:}_{ℜ}s_{t}a_{h}m_{j})$. Similarly, $s_{t}J_{\beta }a_{h}m_{j}⊈N$, and so there exists $b_{\beta }\in J_{\beta }$ such that $s_{t}b_{\beta }a_{h}m_{j}\notin N$ which means that $b_{\beta }\in J_{\beta }-(N{:}_{ℜ}s_{t}a_{h}m_{j})$. As $I_{\alpha }J_{\beta }a_{h}m_{j}\subseteq N$, we obtain that $r_{\alpha }J_{\beta }a_{h}m_{j}\subseteq N$. So, by Lemma 2.15, either $s_{t}r_{\alpha }J_{\beta }m_{j}\subseteq N$, $s_{t}J_{\beta }a_{h}m_{j}\subseteq N$ or $s_{t}r_{\alpha }a_{h}m_{j}\in N$. But $s_{t}r_{\alpha }a_{h}m_{j}\notin N$ and $s_{t}J_{\beta }a_{h}m_{j}⊈N$. So $s_{t}r_{\alpha }J_{\beta }m_{j}\subseteq N$ for all $r_{\alpha }\in I_{\alpha }-(N{:}_{ℜ}s_{t}a_{h}m_{j})$, which means $s_{t}(I_{\alpha }-(N{:}_{ℜ}s_{t}a_{h}m_{j}))J_{\beta }m_{j}\subseteq N$. Since $d_{\beta }\in J_{\beta }$, then $s_{t}r_{\alpha }d_{\beta }m_{j}\in N$. Similarly, as $I_{\alpha }J_{\beta }a_{h}m_{j}\subseteq N$, we get that $I_{\alpha }b_{\beta }a_{h}m_{h}\subseteq N$. This leads, by Lemma 2.15, either $s_{t}I_{\alpha }b_{\beta }m_{j}\subseteq N$, $s_{t}I_{\alpha }a_{h}m_{j}\subseteq N$ or $s_{t}b_{\beta }a_{h}m_{j}\in N$. Since $s_{t}b_{\beta }a_{h}m_{j}\notin N$ and $s_{t}I_{\alpha }a_{h}m_{j}⊈N$, we get $s_{t}I_{\alpha }b_{\beta }m_{j}\subseteq N$ for all $b_{\beta }\in J_{\beta }-(N{:}_{ℜ}s_{t}a_{h}m_{j})$. Hence, $s_{t}I_{\alpha }(J_{\beta }-(N{:}_{ℜ}s_{t}a_{h}m_{j}))m_{j}\subseteq N$. Since $c_{\alpha },r_{\alpha }\in I_{\alpha }$ and $s_{t}I_{\alpha }b_{\beta }m_{j}\subseteq N$, we have $s_{t}c_{\alpha }b_{\beta }m_{j}\in N$ and $s_{t}r_{\alpha }b_{\beta }m_{j}\in N$. Since $(c_{\alpha }+r_{\alpha })\in I_{\alpha }$ and $d_{\beta }+b_{\beta }\in J_{\beta }$, $(c_{\alpha }+r_{\alpha })(d_{\beta }+b_{\beta })a_{h}m_{j}\in N$. Then either $s_{t}(c_{\alpha }+r_{\alpha })a_{h}m_{j}\in N$, $s_{t}(d_{\beta }+b_{\beta })a_{h}m_{j}\in N$ or $s_{t}(c_{\alpha }+r_{\alpha })(d_{\beta }+b_{\beta })m_{j}\in N$. If $s_{t}(c_{\alpha }+r_{\alpha })a_{h}m_{j}=s_{t}c_{\alpha }a_{h}m_{j}+s_{t}r_{\alpha }a_{h}m_{j}\in N$, we get $s_{t}c_{\alpha }a_{h}m_{j}\notin N$ (since $s_{t}r_{\alpha }a_{h}m_{j}\notin N$). This yields that $c_{\alpha }\in$ $I_{\alpha }-(N{:}_{ℜ}s_{t}a_{h}m_{j})$, and so $s_{t}c_{\alpha }d_{\beta }m_{j}\in N$. Similarly, since $s_{t}(d_{\beta }+b_{\beta })a_{h}m_{j}\in N$, we get $s_{t}c_{\alpha }d_{\beta }m_{j}\in N$. If $s_{t}(c_{\alpha }+r_{\alpha })(d_{\beta }+b_{\beta })m_{j}\in N$, then $s_{t}c_{\alpha }d_{\beta }m_{j}+s_{t}c_{\alpha }b_{\beta }m_{j}+s_{t}r_{\alpha }d_{\beta }m_{j}+s_{t}r_{\alpha }b_{\beta }m_{j}r_{\alpha }\in N$. This gives that $s_{t}c_{\alpha }d_{\beta }m_{j}\in N$. Therefore, $s_{t}I_{\alpha }J_{\beta }m_{j}\subseteq N$. □

Theorem 2.17 *Let*B⊆h(ℜ)*be a*m.c.s*of* ℜ *and* $N\leq_{G}ℑ$ *with* $(N{:}_{ℜ}ℑ)\cap B=\emptyset$*. Let* $I={⨁}_{\alpha \in G}I_{\alpha }$*,* $J={⨁}_{\beta \in G}J_{\beta }$ *and* $L={⨁}_{\gamma \in G}L_{\gamma }$ *be graded ideals of* ℜ*, where* $I_{\alpha }=I\cap {ℜ}_{\alpha }$*,* $J_{\beta }=J\cap {ℜ}_{\beta }$ *and* $L_{\gamma }=L\cap {ℜ}_{\gamma }$*. Then the following are equivalent:*

(i) *N is gr-classical B-*2*-absorbing submodule of* ℑ*.*
(ii) *There exists*$s_{t}\in B$*such that for every*α,β,γ∈G*, if*$I_{\alpha }J_{\beta }L_{\gamma }m_{j}\subseteq N$*where*$m_{j}\in h(ℑ)$*and*j∈G*, then either*$s_{t}I_{\alpha }J_{\beta }m_{j}\subseteq N$*or*$s_{t}J_{\beta }L_{\gamma }m_{j}\subseteq N$*or*$s_{t}I_{\alpha }L_{\gamma }m_{j}\subseteq N$*.*

Proof (i)⇒(ii) Suppose that *N* is a *gr*-classical *B*-2-absorbing submodule of ℑ. Let $I_{\alpha }J_{\beta }L_{\gamma }m_{j}\subseteq N$ for some $m_{j}\in h(ℑ)$ and α,β,γ,j∈G. Suppose that $s_{t}I_{\alpha }J_{\beta }m_{j}⊈N$ and $s_{t}I_{\alpha }L_{\gamma }m_{j}⊈N$. We are going to show that $s_{t}J_{\beta }L_{\gamma }m_{j}\subseteq N$. Let $r_{\gamma }\in L_{\gamma }$. Since $s_{t}I_{\alpha }L_{\gamma }m_{j}⊈N$, there is $c_{\gamma }\in L_{\gamma }$ such that $s_{t}I_{\alpha }c_{\gamma }m_{j}⊈N$. As $c_{\gamma },r_{\gamma }\in L_{\gamma }$, we have $c_{\gamma }+r_{\gamma }\in L_{\gamma }$, and so we obtain that $I_{\alpha }J_{\beta }(r_{\gamma }+c_{\gamma })m_{j}\subseteq N$. Thus, by Theorem 2.16, we conclude that either $s_{t}I_{\alpha }(r_{\gamma }+c_{\gamma })m_{j}\subseteq N$ or $s_{t}J_{\beta }(r_{\gamma }+c_{\gamma })m_{j}\subseteq N$. When $s_{t}I_{\alpha }(r_{\gamma }+c_{\gamma })m_{j}\subseteq N$, and since $s_{t}I_{\alpha }c_{\gamma }m_{j}⊈N$, we deduce that $s_{t}I_{\alpha }r_{\gamma }m_{j}⊈N$. Now since $I_{\alpha }J_{\beta }r_{\gamma }m_{j}\subseteq N$, $s_{t}I_{\alpha }J_{\beta }m_{j}⊈N$ and $s_{t}I_{\alpha }r_{\gamma }m_{j}⊈N$, by Theorem 2.16, we reach that $s_{t}J_{\beta }r_{\gamma }m_{j}\subseteq N$. Since $I_{\alpha }J_{\beta }c_{\gamma }m_{j}\subseteq N$, $s_{t}I_{\alpha }J_{\beta }m_{j}⊈N$ and $s_{t}I_{\alpha }c_{\gamma }m_{j}⊈N$, by Theorem 2.16, $s_{t}J_{\beta }c_{\gamma }m_{j}\subseteq N$. Now, when $s_{t}J_{\beta }(r_{\gamma }+c_{\gamma })m_{j}\subseteq N$, then $s_{t}J_{\beta }r_{\gamma }m_{j}\in N$. Therefore, $s_{t}J_{\beta }L_{\gamma }m_{j}\subseteq N$.

(ii)⇒(i) Suppose that (ii) holds. Let abcm∈N where a,b,c∈h(ℜ), m∈h(ℑ). Let I,J and *L* be ideals of ℜ generated by the elements a,b,c respectively, that is I=aR,J=bR and L=cR. So, $I={⊕}_{g\in G}a{ℜ}_{g}$, $J={⊕}_{g\in G}b{ℜ}_{g}$ and $L={⊕}_{g\in G}c{ℜ}_{g}$ are graded ideals of ℜ. Moreover, for every g∈G, $I_{g}=a{ℜ}_{g}$, $J_{g}=b{ℜ}_{g}$, and $L_{g}=c{ℜ}_{g}$. In particular, $I_{e}=a{ℜ}_{e}$, $J_{e}=b{ℜ}_{e}$ and $L_{e}=c{ℜ}_{e}$. Now, by our assumption $I_{e}J_{e}L_{e}m\subseteq N$. Hence, either $s_{t}I_{e}J_{e}m\subseteq N$, $s_{t}J_{e}L_{e}m\subseteq N$ or $s_{t}I_{e}L_{e}m\subseteq N$. So either $s_{t}abm=s_{t}a1b1m\in s_{t}a{ℜ}_{e}b{ℜ}_{e}m=s_{t}I_{e}J_{e}m\subseteq N$, $s_{t}bcm=s_{t}b1c1m\in s_{t}b{ℜ}_{e}c{ℜ}_{e}m=s_{t}J_{e}L_{e}m\subseteq N$ or $s_{t}acm=s_{t}a1c1m\in s_{t}a{ℜ}_{e}c{ℜ}_{e}m=s_{t}I_{e}L_{e}m\subseteq N$. Thus, *N* is *gr*-classical *B*-2-absorbing submodule of ℑ. □

Lemma 2.18 *Let*B⊆h(ℜ)*be a*m.c.s*of* ℜ*. If K is a classical B-*2*-absorbing submodule of* ℑ*, then there exists* $s_{t}\in B$ *such that* $\left(K{:}_{ℑ}s_{t}^{5})=(K{:}_{ℑ}s_{t}^{n}\right)$ *for all* n≥5*.*

Proof Assume that *K* is a *gr*-classical *B*-2-absorbing submodule of ℑ and $s_{t}\in B$ is the element which satisfies the condition of graded classical *B*-2-absorbing submodule. Let $m=\;\sum_{j\in G}m_{j}\in (K{:}_{ℑ}s_{t}^{6})$. Since $\left(K{:}_{ℑ}s_{t}^{6}\right)$ is a graded submodule, we have $m_{j}\in (K{:}_{ℑ}s_{t}^{6})$ for all j∈G. Let j∈G, then $s_{t}^{6}m_{j}=s_{t}^{2}s_{t}^{2}s_{t}^{2}m_{j}\in K$, where $s_{t}^{2}\in h(ℜ)$, hence $m_{j}\in (K{:}_{ℑ}s_{t}^{5})$. So $m=\;\sum_{j\in G}m_{j}\in (K{:}_{ℑ}s_{t}^{5})$. Thus, $\left(K{:}_{ℑ}s_{t}^{6})\subseteq (K{:}_{ℑ}s_{t}^{5}\right)$. Since the other inclusion is always satisfied, we get $\left(K{:}_{ℑ}s_{t}^{5})=(K{:}_{ℑ}s_{t}^{6}\right)$. Now, assume that $\left(K{:}_{ℑ}s_{t}^{5})=(K{:}_{ℑ}s_{t}^{k}\right)$ for all k<n. We will show that $\left(K{:}_{ℑ}s_{t}^{5})=(K{:}_{ℑ}s_{t}^{n}\right)$. Let $m=\;\sum_{i\in G}m_{i}\in (K{:}_{ℑ}s_{t}^{n})$, then $m_{i}\in (K{:}_{ℑ}s_{t}^{n})$ for all i∈G. Let i∈G. Then $s_{t}^{n}m_{i}=s_{t}^{2}s_{t}^{2}s_{t}^{n-4}m_{i}\in K$. Then either $s_{t}s_{t}^{2}s_{t}^{n-4}m_{i}=s_{t}^{n-1}m_{i}\in K$ or $s_{t}s_{t}^{2}s_{t}^{2}m_{i}=s_{t}^{5}m_{i}\in K$. So, we have $m_{i}\in (K{:}_{ℑ}s_{t}^{5})\cup (K{:}_{ℑ}s_{t}^{n-1})$ $=(K{:}_{ℑ}s_{t}^{5})$. Hence, *m*= $\sum_{i\in G}m_{i}\in$ $\left(K{:}_{ℑ}s_{t}^{n})\subseteq (K{:}_{ℑ}s_{t}^{5}\right)$. Thus, $\left(K{:}_{ℑ}s_{t}^{5})=(K{:}_{ℑ}s_{t}^{n}\right)$ for all n≥5. □

Theorem 2.19 *Let*B⊆h(ℜ)*be a*m.c.s*of* ℜ *and* $K\leq_{G}ℑ$ *with* $(K{:}_{ℜ}ℑ)\cap B=\phi$*. Then the following are equivalent:*

(i) *K is gr-classical B-*2*-absorbing submodule of* ℑ*.*
(ii) $\left(K{:}_{ℑ}s_{t}\right)$*is gr-classical* 2*-absorbing submodule of* ℑ *for some* $s_{t}\in B$*.*

Proof (i)⇒(ii) Let *K* be a *gr*-classical *B*-2-absorbing of ℑ. Then there exists $s_{t}\in B$, where t∈G such that whenever $a_{h}b_{i}c_{k}m_{j}\in K$ for some $a_{h},b_{i},c_{k}\in h(ℜ)$, $m_{j}\in h(ℑ)$ and h,i,j,k∈G, then either $s_{t}a_{h}b_{i}m_{j}\in K$, $s_{t}a_{h}c_{k}m_{j}\in K$ or $s_{t}b_{i}c_{k}m_{j}\in K$. By Lemma 2.18, we have $\left(K{:}_{ℑ}s_{t}^{5})=(K{:}_{ℑ}s_{t}^{n}\right)$ for all n≥5. Now our target is to show that $L=(K{:}_{ℑ}s_{t}^{6})=(K{:}_{ℑ}s_{t}^{5})$ is graded classical 2-absorbing submodule of ℑ. Let $a_{h}b_{i}c_{k}m_{j}\in (K{:}_{ℑ}s_{t}^{6})$ for some $a_{h},b_{i},c_{k}\in h(ℜ)$, $m_{j}\in h(ℑ)$ and h,i,j,k∈G. Then $s_{t}^{6}a_{h}b_{i}c_{k}m_{j}=(s_{t}^{2}a_{h})(s_{t}^{2}b_{i})(s_{t}^{2}c_{k})m_{j}\in K$, where $\left(s_{t}^{2}a_{h}),(s_{t}^{2}b_{i}),(s_{t}^{2}c_{k})\in h(ℜ\right)$ and $m_{j}\in h(ℑ)$. Then either $s_{t}^{5}a_{h}b_{i}m_{j}\in K$, $s_{t}^{5}a_{h}c_{k}m_{j}\in K$ or $s_{t}^{5}b_{i}c_{k}m_{j}\in K$. This gives that $a_{h}b_{i}m_{j}\in (K{:}_{ℑ}s_{t}^{5})$, $a_{h}c_{k}m_{j}\in (K{:}_{ℑ}s_{t}^{5})$ or $b_{i}c_{k}m_{j}\in (K{:}_{ℑ}s_{t}^{5})$. Thus, $\left(K{:}_{ℑ}s_{t}^{6}\right)$ is a *gr*-classical 2-absorbing submodule of ℑ.

(ii)⇒(i) Let $a_{h}b_{i}c_{k}m_{j}\in K\subseteq$ $\left(K{:}_{ℑ}s_{t}\right)$ for some $a_{h},b_{i},c_{k}\in h(ℜ)$, $m_{j}\in h(ℑ)$ and h,i,j,k∈G. Since $\left(K{:}_{ℑ}s_{t}\right)$ is *gr*-classical 2-absorbing submodule of ℑ, then either $a_{h}b_{i}m_{j}\in (K{:}_{ℑ}s_{t})$, $a_{h}c_{k}m_{j}\in (K{:}_{ℑ}s_{t})$ or $b_{i}c_{k}m_{j}\in (K{:}_{ℑ}s_{t})$. So $s_{t}a_{h}b_{i}m_{j}\in K$, $s_{t}a_{h}c_{k}m_{j}\in K$ or $s_{t}b_{i}c_{k}m_{j}\in K$. Thus, *K* is *gr*-classical *B*-2-absorbing submodule of ℑ. □

Theorem 2.20 *Let* ℑ*,* ${ℑ}^{\prime }$ *be two graded* ℜ*-modules and* $\psi :ℑ\to {ℑ}^{\prime }$ *be a graded homomorphism. Let* B⊆h(ℜ) *be a* m.c.s *of* ℜ*. If* $K^{\prime }$ *is a gr-classical B-*2*-absorbing submodule of* ${ℑ}^{\prime }$*, then* $\psi^{-1}(K^{\prime })$ *is a gr-classical B-*2*-absorbing submodule of* ℑ*.*

Proof We have $(\psi^{-1}(K^{\prime }){:}_{ℜ}ℑ)\cap B=\emptyset$, because if there exists $s_{g}\in (\psi^{-1}(K^{\prime }){:}_{ℜ}ℑ)\cap B$ with g∈G, then $s_{g}ℑ\subseteq \psi^{-1}(K^{\prime })$. Hence $s_{g}\psi (ℑ)=s_{g}{ℑ}^{\prime }\subseteq K^{\prime }$ and so $s_{g}\in (K^{\prime }{:}_{ℜ}ℑ)$, a contradiction. Now, let $a_{h}b_{i}c_{k}m_{j}\in \psi^{-1}(K^{\prime })$ for some $a_{h},b_{i},c_{k}\in h(ℜ)$, $m_{j}\in h(ℑ)$, and i,j,k,h∈G. Hence $\psi (a_{h}b_{i}c_{k}m_{j})=a_{h}b_{i}c_{k}\psi (m_{j})\in K^{\prime }$. Then there exists $s_{g}\in B$ with g∈G such that $s_{g}a_{h}b_{i}\psi (m_{j})\in K^{\prime }$ or $s_{g}a_{h}c_{k}\psi (m_{j})\in K^{\prime }$ or $s_{g}b_{i}c_{k}\psi (m_{j})\in K^{\prime }$. Thus $\psi (s_{g}a_{h}b_{i}m_{j})\in K^{\prime }$ or $\psi (s_{g}a_{h}c_{k}m_{j})\in K^{\prime }$ or $\psi (s_{g}b_{i}c_{k}m_{j})\in K^{\prime }$. Hence, either $s_{g}a_{h}b_{i}m_{j}\in \psi^{-1}(K^{\prime })$ or $s_{g}a_{h}c_{k}m_{j}\in \psi^{-1}(K^{\prime })$ or $s_{g}b_{i}c_{k}m_{j}\in \psi^{-1}(K^{\prime })$. Thus $\psi^{-1}(K^{\prime })$ is a *gr*-classical *B*-2-absorbing submodule of ℑ. □

Corollary 2.21 *Let*$L\leq_{G}ℑ$*, and*B⊆h(ℜ)*be a*m.c.s*of* ℜ*. If* $K^{\prime }$ *is a gr-classical B-*2*-absorbing submodule of* ℑ *with* $(K^{\prime }{:}_{ℜ}L)\cap B=\emptyset$*, then* $L\cap K^{\prime }$ *is a gr-classical B-*2*-absorbing submodule of L.*

Proof Suppose that $K^{\prime }$ is a *gr*-classical *B*-2-absorbing submodule of ℑ with $(K^{\prime }{:}_{ℜ}L)\cap B=\emptyset$. Consider the graded injection ψ:L→ℑ defined by ψ(m)=m for all m∈L. So $\psi^{-1}(K^{\prime })=L\cap K^{\prime }$. By Theorem 2.20, $L\cap K^{\prime }$ is a *gr*-classical *B*-2-absorbing submodule of *L*. □

Theorem 2.22 *Let* ℑ*,* ${ℑ}^{\prime }$ *be two graded* ℜ*-modules and* $\psi :ℑ\to {ℑ}^{\prime }$ *be a graded epimorphism. Let* B⊆h(ℜ) *be a* m.c.s *of* ℜ*. If K is a gr-classical B-*2*-absorbing submodule of* ℑ *with* ker(f)⊆K*, then* ψ(K) *is a gr-classical B-*2*-absorbing submodule of* ${ℑ}^{\prime }$*.*

Proof We have $(\psi (K){:}_{ℜ}{ℑ}^{\prime })\cap B=\emptyset$, because if there exists $s_{g}\in (\psi (K){:}_{ℜ}{ℑ}^{\prime })\cap B$ where g∈G, then $s_{g}{ℑ}^{\prime }\subseteq \psi (K)$. Thus $\psi (s_{g}M)=s_{g}\psi (ℑ)\subseteq s_{g}{ℑ}^{\prime }\subseteq \psi (K)$. Which implies, $s_{g}ℑ\subseteq s_{g}ℑ+ker(\psi )\subseteq K+ker(\psi )=K$ because ker(ψ)⊆K. Hence $s_{g}ℑ\subseteq K$ and so $s_{g}\in (K{:}_{ℜ}ℑ)$, a contradiction. Now, let $a_{h}b_{i}c_{p}m_{j}^{\prime }\in \psi (K)$ for some $a_{h},b_{i},c_{p}\in h(ℜ)$, $m_{j}^{\prime }\in h({ℑ}^{\prime })$, and i,j,p,h∈G. Then there exists $n_{t}\in K\cap h(ℑ)$ such that $a_{h}b_{i}c_{p}m_{j}^{\prime }=\psi (n_{t})$. Since *ψ* is a graded epimorphism, and $m_{j}^{\prime }\in h({ℑ}^{\prime })$, there exists $m_{j}\in h(ℑ)$ such that $m_{j}^{\prime }=\psi (m_{j})$. Hence $\psi (n_{t})=a_{h}b_{i}c_{k}m_{j}^{\prime }=a_{h}b_{i}c_{p}\psi (m_{j})$, it follows that $n_{t}-a_{h}b_{i}c_{p}m_{j}\in ker(\psi )\subseteq K$ which means $a_{h}b_{i}c_{p}m_{j}\in K$. Then there exists $s_{g}\in B$ such that $s_{g}a_{h}b_{i}m_{j}\in K$ or $s_{g}a_{h}c_{p}m_{j}\in K$ or $s_{g}b_{i}c_{p}m_{k}\in K$. Then $\psi (s_{g}a_{h}b_{i}m_{k})\in \psi (K)$ or $\psi (s_{g}a_{h}c_{p}m_{j})\in \psi (K)$ or $\psi (s_{g}b_{i}c_{p}m_{j})\in \psi (K)$. Then $s_{g}a_{h}b_{i}m_{j}^{\prime }=s_{g}a_{h}b_{i}\psi (m_{j})\in \psi (K)$ or $s_{g}a_{h}c_{p}m_{j}^{\prime }=s_{g}a_{h}c_{p}\psi (m_{j})\in \psi (K)$ or $s_{g}b_{i}c_{p}m_{j}^{\prime }=s_{g}b_{i}c_{p}\psi (m_{j})\in \psi (K)$. Therefore, ψ(K) is a *gr*-classical *B*-2-absorbing submodule. □

Corollary 2.23 *Let*$L\leq_{G}ℑ$*. Then K is a gr-classical B-*2*-absorbing submodules of* ℑ *if and only if* K/L *is a gr-classical B-*2*-absorbing submodule of* ℑ/L*.*

Proof Suppose that *K* is a graded classical *B*-2-absorbing submodule of ℑ. Consider the canonical homomorphism π:ℑ→ℑ/L defined be π(m)=m+L, for all m∈ℑ. Then *π* is a graded epimorphism and ker(π)=L⊆K so by Corollary 2.23, π(K)=K/L is a *gr*-classical *B*-2-absorbing submodule of ℑ/L. Conversely, suppose that K/L is a graded classical *B*-2-absorbing submodule of ℑ/L. Let $a_{h}b_{i}c_{k}m_{j}\in K$ for some $a_{h}$, $b_{i}$, $c_{k}\in h(ℜ)$, $m_{j}\in h(ℑ)$, and i,j,k,h∈G. Then $a_{h}b_{i}c_{k}(m_{j}+L)\in K/L$ where $m_{j}+L\in h(ℑ/L)$. Since K/L is a *gr*-classical *B*-2-absorbing submodule of ℑ/L, then $\exists s_{g}\in B$ where g∈G such that either $(s_{g}a_{h}b_{i}m_{j})+L\in K/L$ or $(s_{g}a_{h}c_{k}m_{j})+L\in K/L$ or $(s_{g}b_{i}c_{k}m_{j})+L\in K/L$. Then either $s_{g}a_{h}b_{i}m_{j}\in K$ or $s_{g}a_{h}c_{k}m_{j}\in K$ or $s_{g}b_{i}c_{k}m_{j}\in K$. Thus *K* is a *gr*-classical *B*-2-absorbing submodule of ℑ. □

## Declarations

### Author contribution statement

Khaldoun Al-Zoubi: Conceived and designed the experiments; Performed the experiments; Analyzed and interpreted the data; Contributed reagents, materials, analysis tools or data; Wrote the paper.

Mohammed Ali: Conceived and designed the experiments; Analyzed and interpreted the data; Wrote the paper.

Mohammad Alkhatib: Analyzed and interpreted the data; Contributed reagents, materials, analysis tools or data; Wrote the paper.

### Funding statement

This research did not receive any specific grant from funding agencies in the public, commercial, or not-for-profit sectors.

### Data availability statement

No data was used for the research described in the article.

### Declaration of interests statement

The authors declare no conflict of interest.

### Additional information

No additional information is available for this paper.
